# Genetic investigation of Nordic patients with complement-mediated kidney diseases

**DOI:** 10.3389/fimmu.2023.1254759

**Published:** 2023-09-07

**Authors:** Viktor Rydberg, Sigridur Sunna Aradottir, Ann-Charlotte Kristoffersson, Naila Svitacheva, Diana Karpman

**Affiliations:** Department of Pediatrics, Clinical Sciences Lund, Lund University, Lund, Sweden

**Keywords:** complement, atypical hemolytic uremic syndrome, C3 glomerulopathy, membranoproliferative glomerulonephritis, genes

## Abstract

**Background:**

Complement activation in atypical hemolytic uremic syndrome (aHUS), C3 glomerulonephropathy (C3G) and immune complex-mediated membranoproliferative glomerulonephritis (IC-MPGN) may be associated with rare genetic variants. Here we describe gene variants in the Swedish and Norwegian populations.

**Methods:**

Patients with these diagnoses (N=141) were referred for genetic screening. Sanger or next-generation sequencing were performed to identify genetic variants in 16 genes associated with these conditions. Nonsynonymous genetic variants are described when they have a minor allele frequency of <1% or were previously reported as being disease-associated.

**Results:**

In patients with aHUS (n=94, one also had IC-MPGN) 68 different genetic variants or deletions were identified in 60 patients, of which 18 were novel. Thirty-two patients had more than one genetic variant. In patients with C3G (n=40) 29 genetic variants, deletions or duplications were identified in 15 patients, of which 9 were novel. Eight patients had more than one variant. In patients with IC-MPGN (n=7) five genetic variants were identified in five patients. Factor H variants were the most frequent in aHUS and C3 variants in C3G. Seventeen variants occurred in more than one condition.

**Conclusion:**

Genetic screening of patients with aHUS, C3G and IC-MPGN is of paramount importance for diagnostics and treatment. In this study, we describe genetic assessment of Nordic patients in which 26 novel variants were found.

## Introduction

Rare genetic variants have been associated with the ultra-rare complement-mediated kidney diseases atypical hemolytic uremic syndrome (aHUS) and C3 glomerulopathy (C3G) as well as the rare kidney disease immune complex-associated membranoproliferative glomerulonephritis (IC-MPGN). HUS is characterized by the simultaneous development of acute kidney injury, non-immune hemolytic anemia, and thrombocytopenia (1). The most common cause of HUS is gastrointestinal infection with Shiga toxin-producing enterohemorrhagic *E. coli* (EHEC) (1). A less common form is aHUS, associated with overactivation of the alternative pathway of complement due to heterozygous variants or the presence of circulating auto-antibodies (2, 3).

In aHUS overactivation of complement has been associated with gain-of-function mutations in complement components such as C3 (4) and factor B (5) or loss-of-function mutations in complement regulators such as factor H (6), factor I (7), and membrane-cofactor protein (MCP or CD46) (8). Rare genetic variants have also been described in factor H-related 5 (9), the terminal complement pathway regulator clusterin (10), thrombomodulin (11), diacylglycerol kinase epsilon (DGKE), often associated with development of HUS in infancy (12), and the fibrinolytic factor plasminogen (13). Variants affecting components of the classical pathway of complement, such as C2 and C4 binding-protein, have also been described (14–16). In addition to specific genetic variants, alone or in combination (17), certain haplotypes in factor H (18) and MCP (19) have been described as being risk-associated for the development of disease. Some patients have antibodies to factor H (3) which may occur in combination with deletions or hybrid genes in factor H-related proteins (20).

aHUS is associated with both familial and sporadic cases (1). The presence of a heterozygous mutation is not necessarily associated with disease. In addition to an underlying genetic variant, found in about 60-70% of cases, a trigger, such as infection or pregnancy, can contribute to the development of aHUS (2). Disease recurrences are common, and the natural course of disease is characterized by the development of end-stage kidney disease which may also recur after transplantation (17).

C3G is a chronic form of glomerulonephritis that can lead to end-stage kidney disease and is further subdivided into C3 glomerulonephritis (C3GN) and Dense Deposit Disease (DDD) based on ultramorphology (21, 22). Complement activation *via* the alternative pathway occurs primarily in the fluid phase (21, 22) either due to mutations or auto-antibodies. This leads to increased C3 consumption, low serum C3 and increased complement deposition in glomeruli (22). Immunofluorescence of kidney biopsies shows predominant C3 deposition and little or no immunoglobulin deposits (23). Patients with C3G carry mutations in *C3* and complement factor B (*CFB*), as well as in complement factor H (*CFH*), complement factor I (*CFI*) and complement factor H-related 5 (*CFHR5*) (24). C3 nephritic factors are auto-antibodies that bind the C3 convertase prolonging its half-life (21, 24) and are found in in many patients (25). Some patients with C3G may have an underlying monoclonal gammopathy (23).

IC-MPGN is also a chronic form of glomerulonephritis that can progress to end-stage renal disease (26). It cannot be differentiated from C3GN based on renal symptoms and ultramorphology but immunofluorescence exhibits deposits of immunoglobulins as well as complement (23, 26). Although IC-MPGN can be associated with infections, such as viral hepatitis, or monoclonal gammopathy (23), cluster analysis revealed that some patients have excessive complement activation *via* the alternative pathway (27), properdin-dependent C3 nephritic factors (28) as well as genetic variants in complement (26). This suggests an overlapping spectrum of disease between C3G and IC-MPGN with regard to etiology and clinical course (26, 29).

Genetic variants have been found in these complement-mediated kidney diseases in several population studies (24, 30–37). The aim of this paper is to describe genetic variants associated with aHUS, C3G and IC-MPGN in patients from Sweden and Norway. Sequencing encompassed the genes encoding complement factor H, C3, factor I, factor B, MCP, C5, factor H-related proteins 1-5, clusterin, DGKE, thrombomodulin, plasminogen, and properdin. Twenty-six novel genetic variants were found, and certain variants were found in more than one condition.

## Methods

### Patients

Patients with suspected complement-mediated renal diseases or thrombotic thrombocytopenic purpura (TTP) are referred to the Dept of Pediatrics, Lund University, for genetic analysis. In this study patients with suspected TTP were not included. A total of 141 patients, both children and adults, living in Sweden or Norway, were included, 94 patients with a clinical diagnosis of aHUS, 40 patients with C3G and 8 patients with IC-MPGN. One patient had both aHUS and IC-MPGN. The diagnosis of aHUS was made by the referring physician based on the simultaneous presentation of non-immune hemolytic anemia, thrombocytopenia, and acute kidney injury as well as negative testing for enterohemorrhagic *E. coli*. Patients with documented defects in cobalamin metabolism were excluded. The diagnosis of C3G and IC-MPGN was based on kidney biopsy results. Patient data consisted of age of disease debut, laboratory data (such as complement levels and the presence of auto-antibodies), course of disease including kidney failure and familial cases. Data regarding treatment was not uniformly available as most samples were collected upon initial diagnosis. Patient data are presented in **Table 1**. Informed consent was obtained for diagnostic genetic analysis and the project was approved by the Swedish Ethical Review Authority, approval no. 2021-04438. The Swedish Ethical Review Authority waived the requirement for written consent from patients included retrospectively in this study. All patients included after October 2021 gave informed written consent.

**Table 1:** Patients investigated in this study.

Diagnosis	Number of patients	Sex	Familial cases
Atypical hemolytic uremic syndrome	94^a^	M: 43 F: 51	14
C3 glomerulopathy	40	M: 18 F: 22	–
Immune complex-mediated membranoproliferative glomerulonephritis	8^a^	M: 4 F: 4	–

M, male; F, female. ^a,^ one patient (# 254 in **Supplementary Tables S1**, **S3**) had both atypical hemolytic uremic syndrome and immune complex-mediated membranoproliferative glomerulonephritis.

### Genetic analysis

Genomic DNA was extracted and analyzed by Sanger sequencing until the end of 2016. These samples were assayed for variants in genes encoding factor H, factor I, membrane cofactor protein CD46, C3 and factor B. From 2017 samples were assayed by next generation sequencing first using whole exome sequencing and from 2020 by whole genome sequencing. A panel of 17 genes was assayed including complement factor H (*CFH*), factor I (*CFI*), membrane cofactor protein (*MCP*, CD46), *C3*, factor B (*CFB*), properdin (*CFP*), clusterin (*CLU*), factor H-related proteins 1-5 (*CFHR1*-*CFHR5*), *ADAMTS13* (a disintegrin and metalloproteinase with a thrombospondin type 1 motif, member 13), thrombomodulin (*THBD*), *DGKE*, *C5* and plasminogen (*PLG*). In this study variants in ADAMTS13 were not included.

For Sanger sequencing genomic DNA was combined with DNA polymerase, primers and nucleotides. The PCR product was sequenced bidirectionally using fluorescent chain-terminating nucleotides (38) from Big dye terminator kit (Applied Biosystems, Foster City, CA) and analyzed on an Applied Biosystems DNA Analyzer, model 3730.

Next generation sequencing was performed in collaboration with the Center for Molecular Diagnostics, Skåne University Hospital, Lund, using either whole exome or whole genome sequencing. Whole-exome sequencing (WES) libraries were generated using Agilent SureSelect Clinical Research Exome v2. Whole-genome sequencing libraries were prepared using Illumina TruSeq PCR-Free. Sequencing was done on either Next Seq 500 (WES) or Novaseq (WES and NGS), with 2 x 150 bp paired end reads and a target depth of at least 30x. Resulting reads were analyzed using the Broad Institute best practices (https://www.broadinstitute.org/gatk/guide/best-practices) as implemented in the Sentieon software suite (https://www.sentieon.com/). Briefly, reads were mapped to the human genome (build hg19) with BWA MEM and variants were identified using DNAscope as implemented in Sentieon. Structural variants were detected with CNVnator and Manta for WGS, and with CNVkit for WES. Variants were annotated using Ensembl Variant Effect Predictor (VEP, https://www.ensembl.org/info/docs/tools/vep/) and filtered for the following genes: *CFH, CFHR1-5, CFI, MCP, CFB, C3, C5, CFP, DGKE, PLG, THBD* and *CLU*. All relevant variants were verified in Integrative Genomics Viewer (IGV, https://software.broadinstitute.org/software/igv). Interpretation of variants was performed using Scout software (Similarities from COntinUous Traits https://clinical-genomics.github.io/scout) and prediction was performed using Mastermind (mastermind.genomenon.com). Certain DNA samples were also sequenced at Centogene, Rostock Germany.

### Data analysis

Sequencing data included nucleotide shift and amino acid alterations as well as zygosity. This information was analyzed using databases describing mutations and polymorphisms in the included genes such as www.complement-db.org, https://gnomad.broadinstitute.org/ and www.ncbi.nlm.nih.gov/snp for variant calling. Minor allele frequency was defined by the frequency of the second most common allele. Minor allele frequency less than 1% was defined as a possible mutation. Variants that were previously described as associated with disease were included even if the minor allele frequency was > 1%. Variants were considered novel if not previously published in the medical literature. Variants that were previously reported in the ClinVar database in association with complement-mediated diseases such as age-related macular degeneration, aHUS or C3G are mentioned.

### Assay of complement and auto-antibodies

C3 was analyzed by nephelometry and C3d by double-decker rocket immunoelectrophoresis according to hospital routines. Low and high levels were defined as below or above the laboratory reference values. Antibodies against factor H and C3 or C4 nephritic factor were detected at the Department of Clinical Immunology, Skåne University Hospital in Lund as per hospital routines. Factor H antibodies were detected as previously described with minor modifications (3). C3 nephritic factor was detected using three methods, by ELISA (39), by hemolytic assay (40) and by crossed immunoelectrophoresis (41) and (42). If any of these assays were positive the patient was defined as having C3 nephritic factor. C4 nephritic factor was detected as previously described (43) with minor modifications.

## Results

### Genetic variants associated with disease

Of all 141 patients with aHUS, C3G and IC-MPGN 80 patients were found to have genetic variants. Of 94 aHUS patients, 60 patients had genetic variants. Of these 32 patients had more than one variant and a total of 68 different aHUS-related variants were identified, considering deletions in *CFHR1* and *CFHR3* as one variant, as these genes are adjacent to each other. The genetic variants in aHUS patients are summarized in **Table 2** and **Supplementary Table 1**. Deletions in *CFHR1* and *CFHR3* are only reported when homozygous except for one patient with antibodies to factor H (patient 17). Eighteen variants were novel to this study and have not been described before. Two of these was previously reported in ClinVar in association with complement-mediated disease. The association between the genetic variants and kidney function, if known, is summarized in **Supplementary Table 1**.

**Table 2:** Variants found in aHUS patients included in this study.

Variant	Nucleotide shift	Type of variant	dbSNP	Domain	Minor Allele frequency	Functional studies	ACMG classification	Reference
CFH								
A48S	c.142G>T	Missense	–	SCR1	Unknown	LoF	VUS	(44)
N516K	c.1548T>A	Missense	rs147403664	SCR9	0.00030409	NPE	VUS	(45, 46)
P621T hom	c.1861C>A	Missense	–	SCR10	0.000007969	LoF	LP	(47)
D693N^a^	c.2077G>A	Missense	rs148403790	SCR12	0.0001592	–	Conflicting	(48)
R830W	c.2488C>T	Missense	rs62641696	SCR14	0.00007826	NPE	VUS	(49)
C870R	c.2608T>C	Missense	rs1221868049	SCR15	0.000003998	LoF	VUS	(50)
Q950H	c.2850G>T	Missense	rs149474608	SCR16	0.003911	LoF or NPE	LP	(45, 51, 52)
N1050Y^b^	c.3148A>T	Missense	rs35274867	SCR18	0.01469	NPE	LB	(45, 53, 54)
I1150M	c.3450A>G	Missense	–	SCR19	Unknown	–	VUS	(55)
H1165D	c.3493C>A	Missense	–	SCR20	Unknown	–	–	This study
V1168E	c.3503T>A	Missense	–	SCR20	Unknown	LoF	–	(50)
S1191L	c.3572C>T	Missense	rs460897	SCR20	0.006394	LoF	P	(45)
S1209T^c^	c.3625T>A	Missense	rs561146868	SCR20	0.00000398	–	LB	(48)
	c.3134-5T>C	Intronic splice	rs513699		0.00016	–	VUS	(56)
C3								
K65Q	c.193A>C	Missense	rs539992721	MG1	0.00004772	GoF	P	(57)
K155Q	c.463A>C	Missense	rs147859257	MG2	0.002705	GoF	LP	(58, 59)
R592W	c.1775G>A	Missense	rs121909583	MG6b	0.000003977	GoF	P	(4)
R735W	c.2203C>T	Missense	rs117793540	ANA	0.002085	NPE	LB	(4)
G1116R	c.3346G>T	Missense	rs138900723	TED	0.000204	–	P	(60)
W1631*	c.4893G>A	Stop	–	C345C	Unknown	–	P	(61)
V1658A	c.4973A>C	Missense	–	C345C	Unknown	GoF	LP	(62)
CFI								
Y206N	c.616T>A	Missense	rs371623439	SRCR	0.00006718	NPE	Conflicting	(63)
G261D	c.782G>A	Missense	rs112534524	LDR2	0.001336	NPE	LB	(64)
S326P hom and heteroz	c.1000T>C	Missense	rs754267987	Linker 2	0.00004377	–	–	This study
G328R	c.1006G>A	Missense	rs144164794	Linker 2	0.00005968	–	LP	(65)
G516V	c.1547G>T	Missense	rs764347930	SP	0.00003586	–	LP	(66)
	c.1534+5G>T	Intronic splice	rs114013791	Intron 12	0.00866	–	B	(33)
CFB								
	c.*47C>T	Intronic	rs375895797	3’UTR	0.0004666	–	Conflicting	This study
T92S^d^	c.274A>T	Missense	rs369638886	SCR 1	0.00006514	–	Conflicting	This study
G252S^b^	c.754G>A	Missense	rs4151651	VWA	0.02245	–	LB	(48)
L433S	c.1298T>C	Missense	–	VWA	0.000407	NPE	LB	(67)
E566A	c.1697A>C	Missense	rs45484591	SP	0.01065	NPE	B	(68)
E601K	c.1801G>A	Missense	rs756325732	SP	0.00001216	NPE	VUS	(69)
D651E	c.1953T>G	Missense	rs4151660	SP	0.002286	**-**	LB	(48)
CD46								
	c.97+1G>A hom	Intronic splice	rs755505712	–	0.00001596	–	–	This study
	c.286+2T>G	Intronic splice	rs769742294	Intron	0.00005205	LoF	P	(70)
Y189D	c.565T>G	Missense	rs202071781	SCR3	0.00001768	LoF	P	(71)
S201L	c.602C>T	Missense	–	SCR 3	Unknown	LoF	LP	(72)
I203T^c^	c.608T>C	Missense	–	SCR 3	0.00017	–	VUS	(48)
T207M	c.620C>T	Missense	rs546619508	SCR 3	0.00002784	–	VUS	This study
	c.947-1G>C	Intronic splice	–	Intron	Unknown	–	–	This study
A353V^b^	c.1013C>T	Missense	rs35366573	TM	0.01541	LoF, NFE	Conflicting	(33, 73, 74,)
C5								
N245S	c.734A>G	Missense	rs201354178	MG3	0.00005946	–	VUS	(75)
I330T	c.989T>C	Missense	rs147430470	MG3	0.0007883	–	Conflicting	This study
E1011D	c.3033G>C	Missense	rs150096192	C5d	0.001124	–	LB	This study
T1298M	c.3893C>T	Missense	rs750246549	C5d/ TED	0.0001061	–	VUS	This study
CFHR1								
Deletion		Deletion				–	–	(76)
CFHR3								
Deletion		Deletion				–	–	(76)
CFHR4								
H447R	c.1340A>G	Missense	rs762274604	SCR7	0.000004186	–	–	This study
D544Ifs*28	c.1626del	Frameshift	rs747535893	SCR9	0.0001063	–	–	This study
	c.1180+2T>C	Intronic splice	–	–	Unknown	–	–	This study
CFHR5								
V110A hom and heteroz	c.329T>C	Missense	rs140691305	SCR2	0.001730	NPE	LB	(77)
E163Kfs*10	c.485_486dup	Frameshift (Insertion)	rs565457964	SCR3	0.006750	NPE	Conflicting	(77)
E163Rfs*35	c.486dup	Frameshift (Insertion)	rs565457964	SCR3	0.002030	**-**	**-**	(77)
E226Dfs*7	c.678del	Deletion	rs1438537910	SCR4	0.000007964	–	P	This study
G278S	c.832G>A	Missense	rs139017763	SCR5	0.007408	NPE	LB	(48)
Y279N	c.835T>A	Missense	rs143240067	SCR5	0.0001274	–	Conflicting	(78)
M514R	c.1541T>G	Missense	rs141321678	SCR9	0.001158	–	Conflicting	(79)
CLU								
E75Q	c.223G>C	Missense	–	α chain	–	–	–	This study
DGKE								
Y326* hom	c.978T>G	Stop codon	rs3748982326	Kinase catalytic domain	Unknown	–	–	This study
S485F hom	c.1454C>T	Missense	–	DAGKa	Unknown	–	–	This study
Q560R	c.1679A>G	Missense	rs61751972	Linker	0.001798	–	VUS	(48)
THBD								
A43T ^c^	c.127G>A	Missense	rs1800576	G type lectin	0.003012	–	VUS	(48)
P228L^a^	c.683C>T	Missense	rs375011249	EGF-1	0.002270	–	–	This study
P499R	c.1496C>G	Missense	rs754426265	EGF-6	0.00003828	–	VUS	(48)^c^
P501L	c.1502C>T	Missense	rs1800579	Linker	0.001785	**-**	VUS	(11)
PLG								
R89K	c.266G>A	Missense	rs143079629	PAN	0.006191	–	B	(48)
R261H	c.782G>A	Missense	rs4252187	Kringle 2	0.002501	–	Conflicting	(80)
S460R	c.1380T>A	Missense	rs116573785	Kringle 4	0.005517	–	B	(80)
G579R^e^	c.1735G>A	Missense	rs138728014	Serine protease	0.0002192	–	VUS	This study

a, Previously reported in the ClinVar database in association with aHUS. b, Minor allele frequency > 1% but this variant was previously associated with aHUS. c, Mentioned in the complement database (www.complement-db.org) with reference to (48). d, Previously reported in the ClinVar database in association with age-related macular degeneration and aHUS. e, The individual bearing this variant did not have aHUS but kidneys were donated postmortem and the recipients developed thrombotic microangiopathy. The individual with this variant was not included in the 94 aHUS patients. CFH, Complement factor H; C3, Complement C3; CFI, Complement factor I; CFB, Complement factor B; CD46, CD46/Membrane cofactor protein; C5, Complement C5; CFHR1-5, Complement factor H related 1-5; CLU, Clusterin; DGKE, Diacylglycerol kinase epsilon; THBD, Thrombomodulin; PLG, Plasminogen; *, Stop codon; Hom, homozygous; Heteroz, heterozygous; SCR, Short consensus repeats; MG1-8, Macroglobulin domain 1-8; ANA, Anaphylatoxin; TED, Thiol ester-containing domain; C345C, C345C/NTR domain; SRCR, Scavenger receptor cysteine-rich; LDR2, LDL receptor class A2; SP, Peptidase S1; VWA, Von Willebrand factor type A; TM, Transmembrane protein; C5d, C5d domain; DAGKa, Diacylglycerol kinase accessory domain; EGF like 6, Epidermal growth factor-like 6; PAN, Plasminogen-Apple-Nematode; LoF, Loss-of-function (including low plasma concentrations); NPE, No phenotypic effect; GoF, Gain-of-function; VUS, Variant of unknown significance; LP, Likely pathogenic; LB, Likely benign; P, Pathogenic; B, Benign.

In 40 C3G patients 15 patients were found to have 29 genetic variants which could be associated with disease (considering homozygous deletions in *CFHR1* and *CFHR3* as one variant). Of these 8 patients had more than one variant. Nine variants in C3G were novel to this study and have not been described before (two of these were previously reported in ClinVar in association with complement-mediated diseases). The genetic variants are summarized in **Table 3**. The association between the genetic variants and kidney function, if known, is summarized in **Supplementary Table 2**.

**Table 3:** Variants in C3G patients included in this study.

Variant or deletion	Nucleotide shift	Type of variant		dbSNP	Domain	Minor Allele frequency	Functional studies			ACMG classification	Reference
CFH											
D693N ^a^	c.2077G>A	Missense		rs148403790	SCR12	0.0001592				Conflicting	(48)
Q950H	c.2850G>T	Missense		rs149474608	SCR16	0.003911	NPE			LP	(45, 51)
N1050Y^b^	c.3148A>T	Missense		rs35274867	SCR18	0.01469	NPE			LB	(45, 54)
S1209T	c.3625T>A	Missense		rs561146868	SCR20	0.00000398	–			LB	(48)
C3											
K155Q	c.463A>C	Missense		rs147859257	MG2	0.002705	GoF			LP	(58, 59)
V326M^c^	c.976G>A	Missense		rs375264020	MG3	0.00004779	–			VUS	This study
Q1061H	c.3183A>T	Missense		rs373054812	TED	0.00007704	–			VUS	This study
E1516A	c.4547A>C	Missense		rs1019532370	C345C	0.00001193	–			VUS	This study
W1631*	c.4893G>A	Stop		NA	C345C	–	LoF			P	(61)
	c.4030-4C>G	Splice acceptor site		NA	Between CUB and MG8	–	–			LB	(55)
CFI											
	c.1534+5G>T	Intronic splice		rs114013791	Intron 12	0.00866	–			–	(33)
G328R	c.981G>A	Missense		rs144164794	Linker 2	–	LoF			LP	(55, 65)
CD46											
A353V^a,b^	c.1013C>T	Missense		rs35366573	TM	0.01541	LoF, NFE			Conflicting	(33, 73)
C5											
P233L	c.698C>T	Missense		rs531284110	MG3	0.0000252	–			VUS	(81)
L354M	c.1060C>A	Missense		rs34552775	MG4	0.0055	–			B	(82)
G385R	c.1153G>C	Missense		–	MG4	Unknown	–			–	This study
CFHR1											
Deletion		Deletion				–	–			LB	(76)
Exon 6 duplication		Duplication					–			LB	This study. Other duplications reported in (83)
CFHR2											
R141S	c.423G>T	Missense		rs142929868	SCR2	0.002947	–			–	This study
CFHR3											
Deletion		Deletion				–	–			–	(76)
CFHR4											
Y43F^d^	c.128A>T	Missense		rs202234955	SCR1	0.001747	–			LB	This study
	c.799+3A>C	Intronic splice		Rs196876631	–	0.001286	–			LB	(82)
CFHR5											
E163Kfs*10	c.485_486dup	Frameshift (insertion)		rs565457964	SCR3	0.006750	NPE			–	(77)
E226Dfs*7	c.678del	Deletion		rs1438537910	SCR4	0.000007964	–			P	This study
Y279N	c.835T>A	Missense		rs143240067	SCR5	0.0001274	–			Conflicting	(78)
R356H^b^	c.1067G>A	Missense		rs35662416	SCR6	0.01633	NPE			LB	(77, 84)
CFP											
D299N	c.895G>A	Missense		rs61737993	TSP t1 5	0.001472	–			B	(85)
CLU											
K444Q	c.1330A>C	Missense	rs2612311022		β-chain	0.0001026	**-**			**-**	This study
PLG											
R89K	c.266G>A	Missense		rs143079629	PAN	0.006191	–			B	(48)
R261H	c.782G>A	Missense		rs4252187	Kringle 2	0.002501	–			Conflicting	(80)

a, Mentioned in the complement database (www.complement-db.org) with reference to (4). b, Minor allele frequency > 1% but this variant was previously associated with aHUS. c, Previously reported in the ClinVar database in association with age-related macular degeneration and aHUS. d, Previously reported in the ClinVar database in association with aHUS. CFH, Complement factor H; C3, Complement C3; CFB, Complement factor B; CFI, Complement factor I; CD46, CD46/Membrane cofactor protein; C5, Complement C5; CFHR1-5, Complement factor H related 1-5; CFP, Complement factor properdin; PLG, Plasminogen. Domains, SCR, Short consensus repeats; MG1-8, Macroglobulin domain 1-8; TED, Thiol ester-containing domain; C345C, C345C/NTR domain; CUB: C1r/C1s, Urchin embryonic growth factor, Bone morphogenetic protein 1; TM, Transmembrane protein; TSP t1, Thrombospondin type-1 1-5; PAN, Plasminogen-Apple-Nematode; NPE, No phenotypic effect; GoF, Gain of function; LOF, Loss of function (including low plasma concentration); VUS, Variant of unknown significance; LP, Likely pathogenic; LB, Likely benign; P, Pathogenic.

Eight patients with IC-MPGN were investigated but one of these also had aHUS (patient 254 in **Supplementary Tables 1**, **3**). In the remaining seven patients, four patients had five genetic variants. The genetic variants are summarized in **Table 4**. The association between the genetic variants and kidney function, if known, is summarized in **Supplementary Table 3**.

**Table 4:** Variants in MPGN-IC patients included in this study.

Variant or deletion	Nucleotide shift	Type of variant	dbSNP	Domain	Minor Allele frequency	Functional studies	ACMG classification	Reference
CFH								
P621T hom	c.1861C>A	Missense	rs762422305	SCR10	0.000007969	LoF	LP	(47)
N1050Y^a^	c.3148A>T	Missense	rs35274867	SCR18	0.01469	NPE	LB	(45, 54)
CD46								
A353V^a^	c.1013C>T	Missense	rs35366573	TM	0.01541	LoF, NFE	Conflicting	(33, 73)
CFB								
I242L	c.724A>C	Missense	rs144812066	Linker	0.001060	NFE	VUS	(30, 69)
THBD								
P501L	c.1502C>T	Missense	rs1800579	Linker	0.001785	-	VUS	(11)
CFHR5								
R356H^a^	c.1067G>A	Missense	rs35662416	SCR6	0.01633	NPE	LB	(77, 84)

a, Minor allele frequency > 1% but this variant was previously associated with aHUS. CFH, Complement factor H Hom, homozygous; CFB, Complement factor B; THBD, Thrombomodulin; Domains, SCR, Short consensus repeats; TM, Transmembrane protein; LoF, Loss of function; NPE, No phenotypic effect; LP, Likely pathogenic; LB, Likely benign; VUS, Variant of unknown significance.

The number of variants found in patients whose DNA underwent Sanger sequencing but did not undergo next generation sequencing may represent an underestimation as only five genes were sequenced encoding CFH, C3, CFI, MCP and CFB.

### Variants in aHUS and C3G

The number of genetic variants detected in patients with aHUS and C3G is summarized in **Tables 5**, **6**, respectively. For patients with IC-MPGN only five variants were detected. In aHUS most variants were detected in the gene encoding CFH followed by C3 and in C3G the reverse was found.

**Table 5:** Prevalence of variants in aHUS patients included in this study (n=94).

Gene	Number of variants	Number of novel variants
*CFH*	14	1
*C3*	7	–
*CFI*	6	1
*CFB*	7	2
*CD46*	8	3
*C5*	4	3
*CFHR1*	1	–
*CFHR3*	1^a^	–
*CFHR4*	3	3
*CFHR5*	7	1
*CLU*	1	1
*DGKE*	3	2
*THBD*	4	1
*PLG*	3	0
**Total**	**68**	**18**

a, A deletion in CFHR1 and CFHR3 was considered one variant as these are neighboring genes and deleted together. CFH: Complement factor H. C3: Complement C3. CFB: Complement factor B. CFI: Complement factor I. CD46: CD46/Membrane cofactor protein. C5: Complement C5. CFHR1-5: Complement factor H related 1-5. PLG: Plasminogen. THBD: Thrombomodulin. DGKE: Diacylglycerol kinase epsilon.

**Table 6:** Prevalence of variants in C3G patients included in this study (n=40).

Gene	Number of variants	Number of novel variants
*CFH*	4	–
*C3*	6	3
*CFI*	2	–
*C5*	3	1
*CD46*	1	–
*CFHR1*	2	1
*CFHR2*	1	1
*CFHR3*	1^a^	–
*CFHR4*	2	1
*CFHR5*	4	1
*CLU*	1	1
*PLG*	2	–
*CFP*	1	–
**Total**	**29**	**9**

a, deletion in CFHR1 and CFHR3 was considered one variant as these are neighboring genes and deleted together. CFH: Complement factor H. C3: Complement C3. CFB: Complement factor B. CFI: Complement factor I. CD46: CD46/Membrane cofactor protein. C5: Complement C5. CFHR1-5: Complement factor H related 1-5. PLG: Plasminogen. CFP: Complement factor properdin.

**Figure 1:**
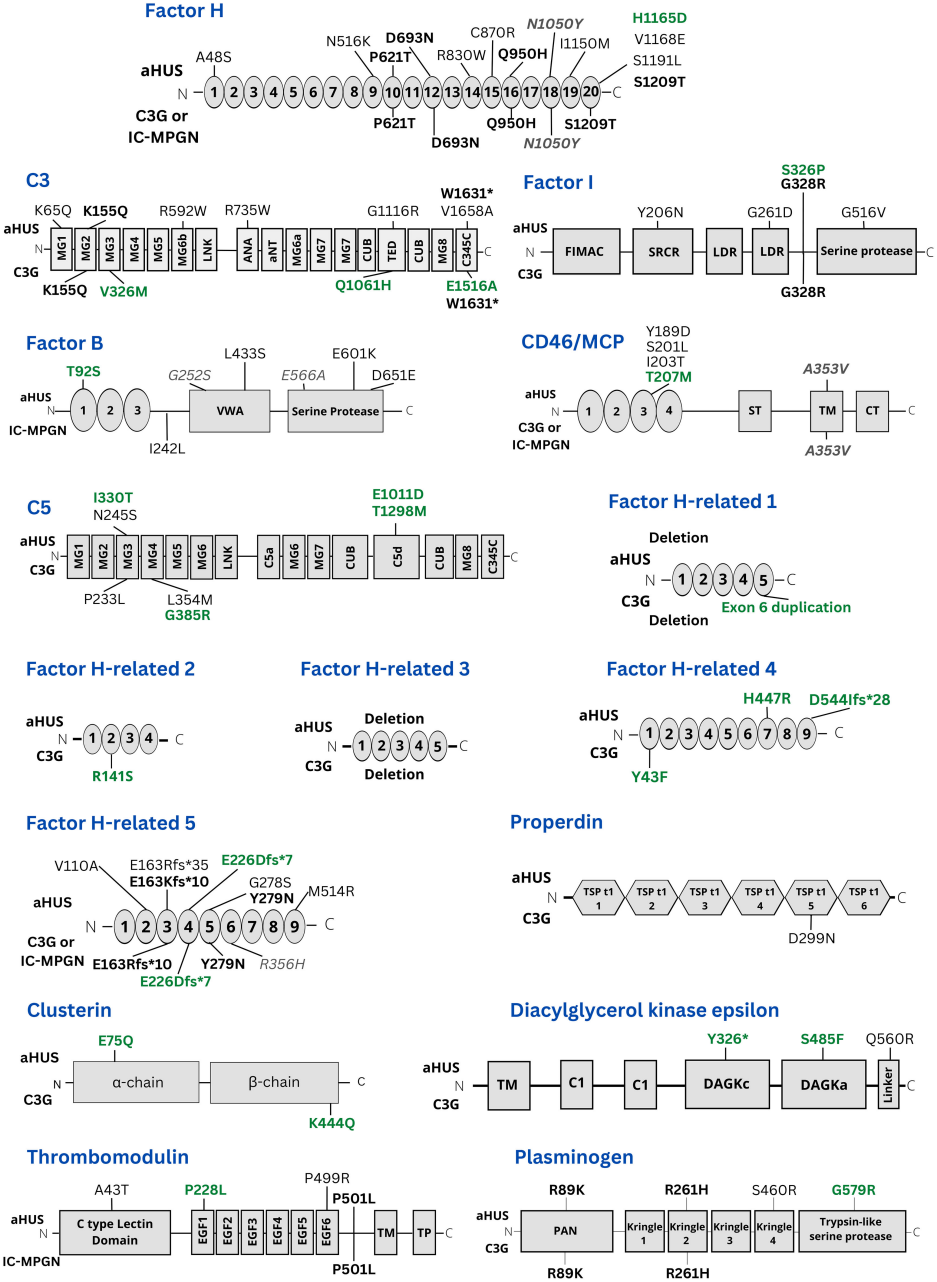
Presentation of all genetic variants found in this study. The location of variants in the genes studied herein is presented. Variants shown above the gene domains were found in aHUS patients. Variants presented under gene domains were found in C3G cases. Variants labeled in black were previously known. Variants labelled in green are novel. Variants in bold were present in both aHUS and C3G patients. Variants in grey italics have a minor allele frequency > 1% but have been associated with complement-mediated kidney diseases. MG1-8: Macroglobulin domain 1-8. ANA: Anaphylatoxin. CUB, C1r/C1s, Urchin embryonic growth factor, Bone morphogenetic protein 1; LNK, Linker; aNT, alpha N-terminal; TED, Thiol ester-containing domain; C345C, C345C/NTR domain; FIMAC, Factor I membrane attack complex; SRCR, Scavenger receptor cysteine-rich; LDR, LDL receptor class A2; VWA, Von Willebrand factor type A; TM, Transmembrane; ST, Ser/Thr-rich; CT, Cytoplasmic domain; EGF 1-6, Epidermal growth factor-like 1-6; PAN, Plasminogen-Apple-Nematode; DAGKc, Diacylglycerol kinase catalytic domain; DAGKa, Diacylglycerol kinase accessory domain; Int, Intron; TSP t1, Thrombospondin type-1 1-5.

The location of all genetic variants detected is presented in **Figure 1** which also depicts which variants are novel and which were found in more than one condition. Most variants were heterozygous, however, homozygous variants were found in *CFH* (P621T), *CFI* (S326P), *CD46* (c.97 + 1G>A), *CFHR5* (V110A), *DGKE* (Y326* and S485F), in patients with aHUS (the patient with the homozygous *CFH* variant P621T also had IC-MPGN). In both aHUS and C3G homozygous deletions in *CFHR1* and *CFHR3* were detected.

Certain variants were found in both aHUS and C3G or IC-MPGN patients. These included *CFH* (P621T in the same patient, D693N, Q950H, N1050Y (the latter with MAF>1%) and S1209T), *C3* (K155Q and W1631*), *CFI* (G328R, c.1534 + 5G>T), *CD46* (A353V, MAF >1%), *CFHR5* (E163Kfs*10, E226DFS*7, Y279N), *THBD* (P501L), *PLG* (R89K and R261H) and homozygous deletions of *CFHR1* and *CFHR3*. Of these *CFHR5* E226DFS*7 was novel and found in both aHUS and C3G patients.

### Genetic variants and the course of disease

Upon referral for genetic testing, we had access to clinical information describing the course of disease in most patients. When these data were available, we correlated the genetic findings to the presence of kidney failure, as presented in **Supplementary Tables 1**-**3**. The genetic variants were correlated to levels of C3, C3d and the presence of antibodies to factor H in aHUS (**Supplementary Table 1**) or nephritic factors in C3G or IC-MPGN (**Supplementary Tables 2**, **3**).

## Discussion

In complement-mediated kidney diseases aHUS, C3G, and IC-MPGN understanding a patient’s genotype and its correlation to disease phenotype is of paramount importance for diagnostics and choice of treatment. Genetic investigation is also crucial for determining the risk of disease recurrence, the suitability of kidney transplantation and the choice of donor, as well as evaluation of the risk of disease development in family members bearing the same variant, including family members considered as kidney donors. This study investigated the genotype of a large cohort of patients with these diseases using a panel of disease-associated genes. In aHUS 68 different variants were identified and in C3G 29 different variants were identified, most variants were heterozygous and 26 were novel. Importantly, 40 patients with aHUS and C3G had more than one genetic variant, exemplifying the complexity of interpreting disease heredity.

Seventeen genetic variants, novel as well as previously reported, had a dual disease phenotype occurring in both patients with aHUS and C3G. The presence of a disease-associated genetic variant is not tantamount to the development of symptoms, as it is known that disease penetrance is incomplete (67, 86) and we show here that identical genetic alterations may be associated with different disease phenotypes. This suggests that disease expression may be related to additional genetic factors (complement genes as well as others), the exposome such as triggering factors and, as yet, undefined environmental, lifestyle or epigenetic factors.

In aHUS and C3G genetic variants were previously identified in complement components and complement regulatory proteins (24, 30–37). The variants may cause a loss-of-function in regulators or a gain-of-function in complement factors. Variants in *CFH* have been shown to lead to a loss-of-function by reducing cofactor activity, as shown for Q950H (52) and V1168E (50), impairing C3b binding, demonstrated for S1191L (87), or by deficiency associated with decreased protein secretion, as shown for P621T (47). Additionally, variant S1191L exhibited impaired ability to regulate complement activation on cell surfaces (87). Genetic variants in factor I can lead to quantitative deficiency or functional defects in protease activity rendering the enzyme incapable of inactivating C3b (59). This was, however, not specifically demonstrated for the variants presented herein, as, for example, the aHUS-associated variant G261D did not exhibit complement dysregulation (64). CD46 variants may also cause loss-of-function by reduced expression on cell surfaces, as shown for c.286 + 2T>G and S201L (70), decreased cofactor activity, or affect C3b/C4b binding capacity (72, 86). Likewise, thrombomodulin inhibits complement activation by promoting C3b inactivation and mutated variants have exhibited less C3b inactivation to iC3b on cell surfaces, as shown for the P501L variant (11).

*C3* and *CFB* variants may exhibit gain-of-function properties in the respective encoded proteins. The variant V1658A in *C3* results in increased C3 convertase formation (62) and K155Q confers increased hemolytic activity (58). Additionally, the C3 variant K65Q is associated with reduced binding to factor H (57), and variant R592W exhibits impaired binding to the complement regulator CD46/MCP (4). *CFB* variants may increase the affinity to C3b, thus stabilizing the convertase, as shown for variant D371G (69) and by increased resistance to factor H-mediated decay (32, 88). However, not all variants show dysfunction in *in vitro* models, as reported for L433S, I242L and E601K (67, 69).

Variants in DGKE cause the development of aHUS in young children, often in combination with proteinuria. It has been suggested that homozygous DGKE variants lead to loss-of-function and consequently a prothrombotic state (12), however the mechanism by which the variants Y326* and S485F lead to disease has yet to be deciphered. Likewise, plasminogen deficiency or dysfunction is associated with reduced proteolytic activity in growing thrombi which may contribute to a thrombosis (89).

Many patients had several variants in more than one gene. In these cases, the individual variants themselves may not lead to disease development, but when combined may result in complement over-activation (67, 90). As previously described in an anephric aHUS patient, with a *CFH* disease-associated haplotype as well as *CFI* and *CFB* variants, the patient had evidence of complement activation, developed carotid artery stenosis, and was successfully treated with eculizumab (67). Some genetic variants are distinctly pathological, even without the presence of other variants, such as the C3 mutation V1636A (62). On the contrary, some variants that *in vitro* exhibit a dysfunctional protein, may be present in unaffected carriers that remain disease-free, such as the *CFH* variant V1168E (50) and the *CFB* variant D371G (91).

For the variants presented herein we provide prediction as to their possible pathogenicity, when available. Prediction models can efficiently assess if a genetic alteration is benign or pathogenic (92), however, they are not always accurate and may require combining various prediction scores (93), thus interpretation can be challenging. Functional tests, using mutant compared to wild-type proteins, can more accurately demonstrate protein dysfunction. Considering the rarity and complexity of disease expression, we recommend performing mutagenesis to predict the effect of genetic variants on complement activation.

This study found that 72% of aHUS and 38% of C3G patients had genetic variants, therefore not all patients who develop disease carry variants in the screened genes. In aHUS this may, in part, be due to the presence of antibodies to factor H and in C3G to the presence of nephritic factors which may cause disease. Additionally, 47 patients were sequenced using only the Sanger method. In these patients, all the known disease-related genes were not sequenced and the percentage of patients with genetic variants may be higher. Even though the frequency of variants in this cohort may be underestimated, other cohorts have identified patients with no known cause for both aHUS and C3G. Previous studies show that about 45-60% of aHUS patients (13, 30, 94) and 30-40% of C3G patients carry mutations (24, 35, 36). Additionally, in C3G and IC-MPGN 50% of patients included herein had C3NeF (or C4NeF) which is comparable to previous reports regarding C3NeF (35, 36). Clearly, all disease-related mechanisms for aHUS and C3G have not been found and future research may uncover new disease-related genes which are not routinely screened for at present.

Patient samples included in this study were collected over a 20-year period. Thus, many patients were included before complement-inhibitory therapy with eculizumab was available. A further limitation of the study is that data regarding treatments was not fully available in referrals, mostly because DNA samples were submitted as part of a diagnostic work-up and, in certain cases, before a diagnosis was made and treatment was initiated. For these reasons, this study could not associate specific genetic variants with the need for complement-inhibitory therapy and outcome.

To conclude, this study presents genetic variants found in Swedish and Norwegian patients with aHUS, C3G and IC-MPGN, 26 of which were novel. Some patients had multiple variants in genes encoding complement proteins. Bearing a genetic variant does not necessarily lead to occurrence of disease as there is incomplete penetrance of the disease phenotype. Furthermore, certain variants were found in both aHUS and C3G suggesting that factors additional to genetic composition can dictate the phenotype. Further investigations are required to better understand the impact variants have on protein functionality, and how they lead to disease expression.

## Data availability statement

All data are available within the article and its supplements. Datasets were deposited in Zenodo, doi: 10.5281/zenodo.8124309. 

## Ethics statement

Informed consent was obtained for diagnostic genetic analysis and the project was approved by the Swedish Ethical Review Authority, approval no. 2021-04438. The Swedish Ethical Review Authority waived the requirement for written consent from patients included retrospectively in this study. All patients included after October 2021 gave informed written consent. The studies were conducted in accordance with the local legislation and institutional requirements. Written informed consent for participation in this study was provided by the participants or their legal guardians/next of kin.

## Author contributions

VR: Formal Analysis, Investigation, Methodology, Writing – original draft, Writing – review & editing. SA: Formal Analysis, Methodology, Writing – original draft, Writing – review & editing. A-CK: Formal Analysis, Methodology, Writing – original draft, Writing – review & editing. NS: Investigation, Writing – original draft. DK: Conceptualization, Formal Analysis, Funding acquisition, Investigation, Methodology, Project administration, Resources, Supervision, Validation, Writing – original draft, Writing – review & editing.
