# Corrigendum: Genetic investigation of Nordic patients with complement-mediated kidney diseases

**DOI:** 10.3389/fimmu.2024.1476204

**Published:** 2024-08-23

**Authors:** Viktor Rydberg, Sigridur Sunna Aradottir, Ann-Charlotte Kristoffersson, Naila Svitacheva, Diana Karpman

**Affiliations:** Department of Pediatrics, Clinical Sciences Lund, Lund University, Lund, Sweden

**Keywords:** complement, atypical hemolytic uremic syndrome, C3 glomerulopathy, membranoproliferative glomerulonephritis, genes

In the published article, there was an error in **Table 3** as published. Under the heading C3, rowc.4030-4C>G was under the ACMG classification stated as “P” when it should be “LB”. Under the heading CFHR2, row R141S, “c.423G>A” should have been written as “c.423G>T”. And finally, under the heading CLU, row K444Q, “c.1339A>C” should be corrected to “c.1330A>C”. The corrected Table 3 and its caption appear below.

**Table 3:** Variants in C3G patients included in this study.

Variant or deletion	Nucleotide shift	Type of variant		dbSNP	Domain	Minor Allele frequency	Functional studies			ACMG classification	Reference
CFH											
D693N ^a^	c.2077G>A	Missense		rs148403790	SCR12	0.0001592				Conflicting	(48)
Q950H	c.2850G>T	Missense		rs149474608	SCR16	0.003911	NPE			LP	(45, 51)
N1050Y^b^	c.3148A>T	Missense		rs35274867	SCR18	0.01469	NPE			LB	(45, 54)
S1209T	c.3625T>A	Missense		rs561146868	SCR20	0.00000398	–			LB	(48)
C3											
K155Q	c.463A>C	Missense		rs147859257	MG2	0.002705	GoF			LP	(58, 59)
V326M^c^	c.976G>A	Missense		rs375264020	MG3	0.00004779	–			VUS	This study
Q1061H	c.3183A>T	Missense		rs373054812	TED	0.00007704	–			VUS	This study
E1516A	c.4547A>C	Missense		rs1019532370	C345C	0.00001193	–			VUS	This study
W1631*	c.4893G>A	Stop		NA	C345C	–	LoF			P	(61)
	c.4030-4C>G	Splice acceptor site		NA	Between CUB and MG8	–	–			LB	(55)
CFI											
	c.1534+5G>T	Intronic splice		rs114013791	Intron 12	0.00866	–			–	(33)
G328R	c.981G>A	Missense		rs144164794	Linker 2	–	LoF			LP	(55, 65)
CD46											
A353V^a,b^	c.1013C>T	Missense		rs35366573	TM	0.01541	LoF, NFE			Conflicting	(33, 73)
C5											
P233L	c.698C>T	Missense		rs531284110	MG3	0.0000252	–			VUS	(81)
L354M	c.1060C>A	Missense		rs34552775	MG4	0.0055	–			B	(82)
G385R	c.1153G>C	Missense		–	MG4	Unknown	–			–	This study
CFHR1											
Deletion		Deletion				–	–			LB	(76)
Exon 6 duplication		Duplication					–			LB	This study. Other duplications reported in (83)
CFHR2											
R141S	c.423G>T	Missense		rs142929868	SCR2	0.002947	–			–	This study
CFHR3											
Deletion		Deletion				–	–			–	(76)
CFHR4											
Y43F^d^	c.128A>T	Missense		rs202234955	SCR1	0.001747	–			LB	This study
	c.799+3A>C	Intronic splice		Rs196876631	–	0.001286	–			LB	(82)
CFHR5											
E163Kfs*10	c.485_486dup	Frameshift (insertion)		rs565457964	SCR3	0.006750	NPE			–	(77)
E226Dfs*7	c.678del	Deletion		rs1438537910	SCR4	0.000007964	–			P	This study
Y279N	c.835T>A	Missense		rs143240067	SCR5	0.0001274	–			Conflicting	(78)
R356H^b^	c.1067G>A	Missense		rs35662416	SCR6	0.01633	NPE			LB	(77, 84)
CFP											
D299N	c.895G>A	Missense		rs61737993	TSP t1 5	0.001472	–			B	(85)
CLU											
K444Q	c.1330A>C	Missense	rs2612311022		β-chain	0.0001026	**-**			**-**	This study
PLG											
R89K	c.266G>A	Missense		rs143079629	PAN	0.006191	–			B	(48)
R261H	c.782G>A	Missense		rs4252187	Kringle 2	0.002501	–			Conflicting	(80)

a, Mentioned in the complement database (www.complement-db.org) with reference to (4). b, Minor allele frequency > 1% but this variant was previously associated with aHUS. c, Previously reported in the ClinVar database in association with age-related macular degeneration and aHUS. d, Previously reported in the ClinVar database in association with aHUS. CFH, Complement factor H; C3, Complement C3; CFB, Complement factor B; CFI, Complement factor I; CD46, CD46/Membrane cofactor protein; C5, Complement C5; CFHR1-5, Complement factor H related 1-5; CFP, Complement factor properdin; PLG, Plasminogen. Domains, SCR, Short consensus repeats; MG1-8, Macroglobulin domain 1-8; TED, Thiol ester-containing domain; C345C, C345C/NTR domain; CUB: C1r/C1s, Urchin embryonic growth factor, Bone morphogenetic protein 1; TM, Transmembrane protein; TSP t1, Thrombospondin type-1 1-5; PAN, Plasminogen-Apple-Nematode; NPE, No phenotypic effect; GoF, Gain of function; LOF, Loss of function (including low plasma concentration); VUS, Variant of unknown significance; LP, Likely pathogenic; LB, Likely benign; P, Pathogenic.

In the published article, there was an error in Supplementary Table 1. The C3 level of patient 314 was given as “normal” when it should have been written as “low”. The corrected Supplementary Material File has now been published.

The authors apologize for these errors and state that they do not change the scientific conclusions of the article in any way. The original article has been updated.

